# Impaired Musculoskeletal Response to Age and Exercise in PPARβ^−/−^ Diabetic Mice

**DOI:** 10.1210/en.2014-1585

**Published:** 2014-10-03

**Authors:** He Fu, Beatrice Desvergne, Serge Ferrari, Nicolas Bonnet

**Affiliations:** Division of Bone Diseases (S.F., N.B.), Department of Internal Medicine Specialties, Geneva University Hospital, and Faculty of Medicine, Geneva 14, CH-1211 Geneva, Switzerland; and Center of Integrative Genomics (H.F., B.D.), Genopode, Lausanne Faculty of Biology and Medicine, CH-1015 Lausanne, Switzerland

## Abstract

Fragility fractures are recognized complication of diabetes, but yet the underlying mechanisms remain poorly understood. This is particularly pronounced in type 2 diabetes in which the propensity to fall is increased but bone mass is not necessarily low. Thus, whether factors implicated in the development of insulin resistance and diabetes directly impact on the musculoskeletal system remains to be investigated. *PPAR*β^−/−^ mice have reduced metabolic activity and are glucose intolerant. We examined changes in bone and muscle in *PPAR*β^−/−^ mice and investigated both the mechanism behind those changes with age as well as their response to exercise. Compared with their wild type, *PPAR*β^−/−^ mice had an accelerated and parallel decline in both muscle and bone strength with age. These changes were accompanied by increased myostatin expression, low bone formation, and increased resorption. In addition, mesenchymal cells from *PPAR*β^−/−^ had a reduced proliferation capacity and appeared to differentiate into more of an adipogenic phenotype. Concomitantly we observed an increased expression of PPARγ, characteristic of adipocytes. The anabolic responses of muscle and bone to exercise were also diminished in *PPAR*β^−/−^ mice. The periosteal bone formation response to direct bone compression was, however, maintained, indicating that PPARβ controls periosteal bone formation through muscle contraction and/or metabolism. Taken together, these data indicate that PPARβ deficiency leads to glucose intolerance, decreased muscle function, and reduced bone strength. On a molecular level, PPARβ appears to regulate myostatin and PPARγ expression in muscle and bone, thereby providing potential new targets to reverse bone fragility in patients with metabolic disturbances.

Several cohort studies and meta-analyses have demonstrated that people suffering from type 2 diabetes mellitus (T2DM) have a higher risk of fracture despite their higher-than-average bone mineral density (BMD) (1–3). The surprisingly higher incidence of fracture in T2DM may be explained by both a decrease in bone quality and an increased propensity to fall. Recent investigations in humans have indicated there is a negative alteration of the trabecular and cortical microarchitecture as well as the bone material properties, ie, components of bone quality, in patients with T2DM (4, 5). There are several potential mechanisms for reduced bone quality in diabetic patients, including reduced bone formation, increased bone resorption, collagen modifications by advanced glycation end products, and lipid accumulation in the bone marrow (6). In addition, there are a number of mechanisms by which diabetes may contribute to falls, including peripheral neuropathy and decreased muscle strength (7, 8). Interestingly, all T2DM features such as insulin resistance and reduced muscle strength have been associated with low levels of physical activity (9), suggesting that exercise training itself may be an efficient intervention in the management of T2DM. Several rodent models have been developed and/or are currently being investigated to help elucidate the pathophysiology of bone fragility in diabetes; however, most of these models have inherent limitations so that the molecular mechanisms that may link alterations in glucose and bone metabolism to muscle and bone alterations remain unclear (10). In particular, the molecular mechanisms for the potentially impaired musculoskeletal response to exercise have not been investigated.

Transcription factors of the peroxisome proliferator-activated receptor (PPAR) family (PPARα, PPARβ, PPARγ) are known to play an important role in energy balance. PPARγ and more recently PPARβ have been shown to be implicated in metabolic dysregulation. More precisely, a decrease in PPARβ expression results in decreased glycolysis and lipogenesis in the liver while increasing fat infiltration in skeletal muscle (11–13). Thus, *PPAR*β^−/−^ mice are metabolically less active and develop glucose intolerance (11). Conversely, activation of PPARβ genetically or pharmacologically promotes muscle oxidative capability by increasing the number of mitochondria formed in the cell, up-regulating myoglobulin, and increasing the proportion of type I (oxidative or slow twitch) muscle fibers (15–17). Hence, sufficient PPARβ expression positively modulates the capacity to perform physical activity, improves insulin sensitivity, and leads to reduced adiposity. It is not surprising therefore that a PPARβ agonist has been used in an animal model to successfully treat insulin resistance (18).

In addition to their lower muscle mass, reduced power, and resistance (15, 16), *PPAR*β^−/−^ mice have recently been reported to have increased bone turnover and decreased bone mass (20). This observation implicates PPARβ in the pathogenesis of bone fragility observed in diabetes. This work showed that in the absence of *PPAR*β, Wnt-β-catenin signaling was altered and associated with lower osteoprotegerin, higher osteoclast number, and osteopenia. However, the function of *PPAR*β on bone formation, particularly bone modeling, and its role in the bone response to exercise is yet to be elucidated. Here we characterize alterations in the biomechanical responses of bones from *PPAR*β^−/−^ mice and identify new molecular targets, namely PPARγ and myostatin, that may contribute to the impairment of the muscle and bone functions in diabetes.

## Materials and Methods

### Animals

*PPAR*β^−/−^ mice were generated from a mixed background (Sv129/C57BL/6) and have been previously described (21). Mice were housed five per cage, maintained under standard nonbarrier conditions, and had access to water and soft diet ad libitum (Harlan Teklad 2019, SDS). The mice were maintained at 22°C with a12-hour light, 12-hour dark cycle. A first group of mice was used to describe the role of PPARβ on bone and muscle phenotype in growing, young adult, adult, and old mice from 1 to 18 months old (n = 8 female mice/group); a second group of 12-week-old male mice were used for the treadmill exercise (n = 8 male mice/group), and a supplemental group of male mice aged 14 weeks were used for the axial compression studies (n = 6 male mice/group). To measure the dynamic indices of bone formation, mice received sc injections of calcein 9 and 2 days before euthanasia.

The femurs, tibiae, and vertebrae were excised and cleared of fat and connective tissues. Bone and muscle tissue that were to be used for RNA analysis were freshly extracted and frozen on liquid nitrogen. Bones for histomorphometry analysis were immediately fixed in 10% formaldehyde for 48 hours at +4°C. Other bones were placed in damp cloth and frozen at −20°C for subsequent microarchitectural and biomechanical tests. All animal procedures were designed in accordance with the Swiss Federal Act on Animal Protection, approved by the University of Geneva School of Medicine Ethical Committee and the State of Geneva Veterinarian Office.

### Glucose tolerance tests

Glucose tolerance tests were performed after a 6-hour fast by measuring blood glucose on glucose strips and an Accu-check glucometer (Roche) at baseline and at 15, 30, 60, 90, and 120 minutes after an ip injection of glucose at 2 g/kg body weight. Blood was obtained after an incision at the base of the tail.

### Handgrip test and locomotor activity

In vivo hindlimb grip strength was measured using an automated grip strength (Bioseb; In Vivo Research Instruments) as previously described (22). Each mouse was tested five times with a 40-second rest interval between tests. The average peak tension and the best attempts were used as a gauge of muscle strength. Two locomotor tests were performed using a treadmill to evaluate both endurance and aerobic maximum speed. For the evaluation of endurance, mice from each group were placed on the treadmill, submitted to moderate exercise at a speed of 14 m/min, and were left to run for as long as they could (mice were considered fatigued when they touched the back of the treadmill five times). The collected end point data were the distance and the time run by each mouse. For the evaluation of aerobic maximum speed, exercise workloads were selected to gradually progress in increments from moderate to maximal intensity. The exercise began with 5 minutes of exercise at a speed of 8 m/min, and speed was thereafter increased by 2 m/min every 2 minutes. The maximal speed was presented as the aerobic maximum speed.

### Exercise training

Twelve-week-old male mice were randomly allocated into sedentary and exercise-trained groups for each genotype: *PPAR*β^−/−^ and *PPAR*β^+/+^ mice. The mice were trained 5 days per week for 6 weeks. The exercise protocol was determined and adapted to the maximal capacity of *PPAR*β^−/−^ mice to perform an exercise on a treadmill: at a speed of 16 m/min, for 30 minutes, with an inclination of 5%. During the first acclimatization week, the treadmill speed and the duration of each running session were gradually increased from 8 m/min for 10 minutes to 16 m/min for 30 minutes. For the last 5 weeks, the running sessions consisted of 16 m/min for 30 minutes with a treadmill inclination of 8°, corresponding to moderate exercise at this specific age (16).

### In vivo axial compression

Fourteen-week-old male mice were subjected to axial compression stimulation on 3 alternate days per week for 2 weeks. The loading apparatus used had been specifically adapted for mice tibiae as previously described (23). Mice were anesthetized by isoflurane 2%, and the tibiae were placed on the stimulation machine between the moving pad on the proximal side (the knee) and the fixed pad on the distal side (the foot). The left tibia of each mouse was subjected to dynamic axial stimulation, using the following parameters: peak load = 12 N; peak strain (midshaft cortex) = 1500 μϵ; pulse period (trapeze shaped pulse) = 0.1 second; rest time between pulses = 10 seconds; and full cycle frequency (pulse + rest) = 0.1 Hz. A total of 40 cycles (∼7 min) were applied per session. The nonstimulated right tibia served as an internal control.

### In vivo measurement of body composition and BMD

Body composition and total body, femoral, and spinal BMD (grams per square centimeter) were measured in vivo by dual-energy X-ray absorptiometry (PIXImus2; GE Lunar) at 12 and 18 weeks of age in the exercise experiment; at 13 and 16 weeks of age in the axial compression experiment; and at 1, 4, 12, and 18 months for the aging experiment. Lean limb mass was evaluated by positioning the region of interest perpendicular to the vertebral column and with the corner of the region of interest aligned to the anterior margin of the hip.

### Ex vivo measurement of morphology and microarchitecture

Microcomputed tomography (UCT40; Scanco Medical AG) was used to assess the trabecular microarchitecture in the excised fifth lumber spine body and distal femur and cortical bone geometry at the midshaft femoral diaphysis as previously described (24). Trabecular and cortical bone regions were evaluated using an isotropic voxel resolution of 12 μm. For the vertebral trabecular region, we evaluated 250 transverse computed tomography slices between the cranial and caudal end plates, excluding 100 μm near each endplate. For the femoral and tibial trabecular regions, to eliminate the primary spongiosa, we analyzed 100 slices from the 50 slices under the distal growth plate. Femoral cortical geometry was assessed using 50 continuous computed tomography slides (600 μm) located at the femoral midshaft. For the trabecular bone regions, we assessed the bone volume fraction (BV/TV, percentage), trabecular thickness (micrometers), trabecular number (millimeters^−1^), trabecular connectivity density (millimeters^−3^), and structural model index. For the cortical bone at the femoral and tibial midshaft, we measured the cortical tissue volume (CtTV; cubic millimeters), cortical bone volume (CtBV; cubic millimeters), the marrow volume (cubic millimeters), and the average cortical width (micrometers). To evaluate bone marrow adiposity tibias, we processed protocol with osmium staining as described (25). After labeling of lipids by osmium tetroxide, the bones were imaged using energy of 45 keV.

### Testing of mechanical resistance

The night before the mechanical testing, bones were thawed slowly at 7°C and then maintained at room temperature. A three-point bending test was performed on the femur as follows: bones were placed on the material testing machine on two supports separated by a distance of 9.9 mm, and the load was applied to the midpoint of the shaft. The mechanical resistance to failure was tested using a servocontrolled electromechanical system (Instron 1114; Instron Corp) with the actuator displaced at 2 mm/min. Outcomes measured were ultimate force, stiffness, and energy as described previously (26).

### Histomorphometry

After 48 hours of fixation, bones were dehydrated in absolute acetone and embedded in methyl-methacrylate (Merck), and 8-μm-thick transversal sections of the midshaft were cut with a Leica Polycut E microtome (Leica Corp Microsystems AG) and mounted unstained for the evaluation of fluorescence. Five-micrometer-thick sagittal sections were stained with modified Goldner's trichrome, and histomorphometric measurements were performed on the secondary spongiosa of the proximal tibia metaphysis and on the endocortical and periosteal bone surfaces in the middle of the tibia using a Leica Q image analyzer (Leica Corp) at ×40 magnification. All parameters were calculated and expressed according to standard formulas and nomenclatures (27): mineral apposition rate (micrometers per day), mineralizing surface per bone surface (percentage), bone formation rate (cubic micrometers per square micrometer per day). Osteoclast surface per bone surface and numbers were evaluated only at 4 and 18 months of age.

### RNA extraction and quantitative PCR

The whole tibia was excised, and both tibial extremities were cut to remove the bone marrow from the diaphysis by flushing with cold PBS. Muscle gastrocnemius was frozen by dipping them on liquid nitrogen. Muscle and tibial diaphysis plus extremities were immediately pulverized to a fine powder and homogenized in peqGold Trifast (peQLab Biotechnologie GmbH) using FastPrep system apparatus (QBiogene). Total RNA was extracted and then purified on minicolumns (RNeasy minikit; QIAGEN) in combination with a deoxyribonuclease treatment (ribonuclease free deoxyribonuclease set; QIAGEN) to avoid DNA contamination. Single-stranded cDNA templates for quantitative real-time PCR analyses were carried out using SuperScript III reverse transcriptase (Invitrogen AG) following the manufacturer's instructions. Quantitative RT-PCR was performed using predesigned TaqMan gene expression assays (references in Supplemental Material). Relative quantities were calculated with the formula, relative quantity = E − cycle threshold using an efficiency (E) of 2 by default. For each gene the mean quantity was calculated from triplicates for each sample, and this quantity was normalized to the similarly measured mean quantity of the glyceraldehyde-3-phosphate dehydrogenase normalization gene. Finally, normalized quantities were averaged for three to four animals.

### Primary osteoblast culture

Primary osteoblast cultures were isolated from *PPAR*β^−/−^ and *PPAR*β^+/+^ newborn calvaria in a medium permissive to mineralization. For this purpose, cells were harvested by sequential collagenase type II (3 mg/mL; Sigma-Aldrich) digestions of calvaria from 2- to 3-day-old *PPAR*β^+/+^ or *PPAR*β^−/−^ mice, half issued from male and half from female pups. Cells from the third to fifth digestions were pooled and cultured in αMEM (Gibco), supplemented with 10% fetal calf serum (Amimed), antibiotics (penicillin 100 U/ml, streptomycin 100 μg/mL; Gibco), glutamine (200 mM; Gibco), amino acids (Amimed), and amphotericin B (0.25 μg/mL; Amimed). Parameters measured included cell proliferation, differentiation, and relative gene expression of osteoblast and osteocytic markers (details are described in Supplemental Material).

### Bone marrow mesenchymal stem cell enriched culture

Mesenchymal stem cells (MSCs) were isolated from the femurs of *PPAR*β^−/−^ and *PPAR*β^+/+^ adult mice. For this purpose, after removing soft tissue and epiphysis, bone marrow was flushed with DMEM medium. Marrow was then submitted to a 15-min enzymatic digestion. Collagenase activity was neutralized and marrow was then resuspended in αMEM (Gibco) supplemented with 10% fetal calf serum (Amimed), antibiotics (penicillin 100 U/mL, streptomycin 100 μg/mL; Gibco), glutamine (200 mM; Gibco), amino acids (Amimed), and amphotericin B (0.25 μg/mL; Amimed). The cells were plated at 10^6^/cm^3^ cells in a T75 flask and incubated at 37°C with 5% CO_2_, and the media were changed every 3 days until the cells reached 80% confluence. Outcomes were the quantification of MSC *PPAR*β^−/−^ and *PPAR*β^+/+^ differentiation into adipocyte and osteoblast populations. For adipogenesis, when MSCs reached 100% of confluence, adipogenesic media (DMEM with 10% fetal bovine serum, 1 μm dexamethasone, 0.5 mm isobutylmethylxanthine, 10 μg/mL insulin, and 100 μm indomethacin) were added. After 3 days of the adipogenic medium, cells were returned to a medium with DMEM, l0% fetal calf serum, l μm dexamethasone, and 5 pg/mL insulin that was refreshed twice per week. After 14 days in culture, adipocytes were visualized by oil red staining and quantified at 540 nm on a spectrophotometer. For osteoblastogenesis, when the MSCs reached 100% of confluence, osteogenic media (DMEM with 10% fetal bovine serum, 10% 100 mM β-glycerophosphate, 10^−8^ M dexamethasone, 5 mg ascorbic acid-2 phosphate) were added and replaced twice per week. After 14 days in culture, calcium depositions were visualized by alizarin red S staining and quantified at 405 nm on a spectrophotometer.

### Collection of serum

Blood from all mice was obtained by a submandibular collection at 1 and 18 months of age. After centrifugation, serum was removed and stored at −80°C until analysis. Serum carboxy-terminal collagen cross-links and osteocalcin were measured according to the manufacturer's instructions (SBA Sciences and Biomedical Technologies Inc). Myostatin was measured in serum by an ELISA with a kit from United States Biological Corp) following the manufacturer's instructions.

### Data analysis

We first tested the effects of exercise or loading within groups (*PPAR*β^−/−^ and *PPAR*β^+/+^) by paired or unpaired *t* tests. In the mechanical loading experiments, we compared stimulated and nonstimulated tibia in the same animal using a paired *t* test. For the exercise investigation, we compared sedentary vs trained animals using unpaired *t* tests. To compare the effect of genotype and the response to loading (mechanical loading and exercise), we used a two-way ANOVA. As appropriate, post hoc testing was performed using Fisher's protected least squares difference. The *P* value of interaction between the genotype and loading (mechanical stimulation or exercise) was mentioned only when it was found to be significant. We then tested the effects of repeated measures within groups (for the effects of aging) by a one-way repeated-measures ANOVA with the genotype used as a factor. Differences were considered significant at *P* ≤ .05. Data are presented as mean ± SEM.

## Results

### Body weight, lean mass, muscle strength, and BMD

First, we confirmed that *PPAR*β^−/−^ mice were glucose intolerant at 4 and 18 months and found that this alteration was maintained, but not exacerbated, as they age (Supplemental Figure 1A). We found the same trend at the age of 12 months but the difference was not statistically significant.

**Figure 1.**
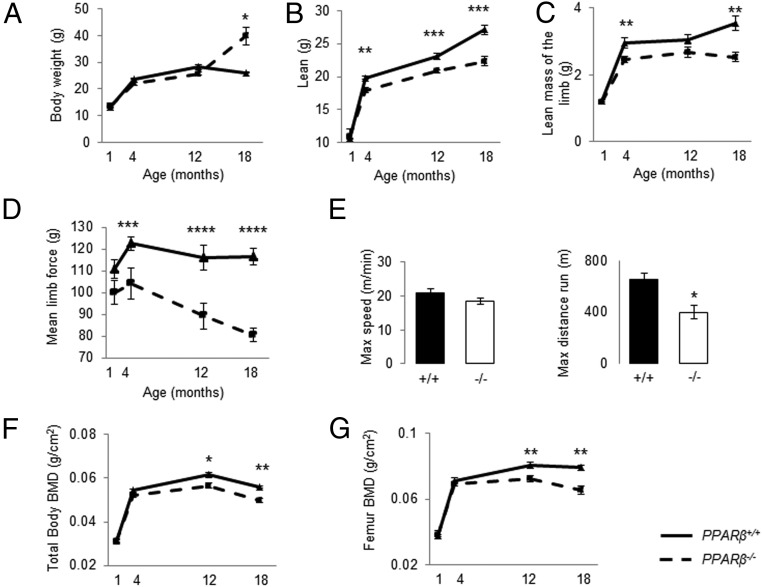
Effect of aging in the absence of PPARβ on body composition, muscle mass, and strength. A, Body weight. B and C, Lean mass of the total body and limb evaluated by PIXImus (GE Lunar). D, Mean force of the limb evaluated by handgrip. E, Maximal speed and distance evaluated on a treadmill at 18 months of age. F and G, Total body and femur BMD. *, *P* < .05; **, *P* < .01; ***, *P* < .001; ****, *P* < .0001 significant difference vs *PPAR*β^−/−^ mice. Continuous lines, *PPAR*β^+/+^; hatched lines, *PPAR*β^−/−^. Bars show means (±SEM). Closed bars, *PPAR*β^+/+^; open bars, *PPAR*β^−/−^.

At 1 month of age, body weight and lean mass did not significantly differ between *PPAR*β^−/−^ and *PPAR*β^+/+^ mice, indicating that PPARβ deficiency did not affect embryonic or early postnatal development. Between 1 and 12 months of age, total and limb lean mass gains were lower in *PPAR*β^−/−^ vs *PPAR*β^+/+^ (Figure 1, B and C), and muscle strength declined in *PPAR*β^−/−^, whereas it remained stable for *PPAR*β^+/+^ (Figure 1D). More specifically, gastrocnemius muscle mass became significantly lower with age in *PPAR*β^−/−^ vs *PPAR*β^+/+^ mice (−4.6% at 4 months and −30.8% at 18 months of age, *P* < .05). Maximal running distance was significantly reduced in old *PPAR*β^−/−^ vs *PPAR*β^+/+^ mice, whereas the maximal speed was unchanged (Figure 1E). Moreover, quantitative real-time PCR performed on RNA from the gastrocnemius indicated lower *Cyclin D1*, *Mip*, *Hif*, *Foxo3*, *mp2*, and *BMP3* and higher *Mstn* expression in *PPAR*β^−/−^ compared with *PPAR*β^+/+^ mice (Supplemental Figure 2). IGF-1 expression was not significantly different between *PPAR*β^−/−^ and *PPAR*β^+/+^ mice. BMD gain was unaffected by PPARβ deficiency until 4 months of age. However, by 12 months of age, total body and femur BMDs were significantly lower in *PPAR*β^−/−^ vs *PPAR*β^+/+^ (Figure 1, F and G), paralleling the alterations in muscle mass/function.

### Bone microarchitecture, turnover, and biomechanical properties

**Figure 2.**
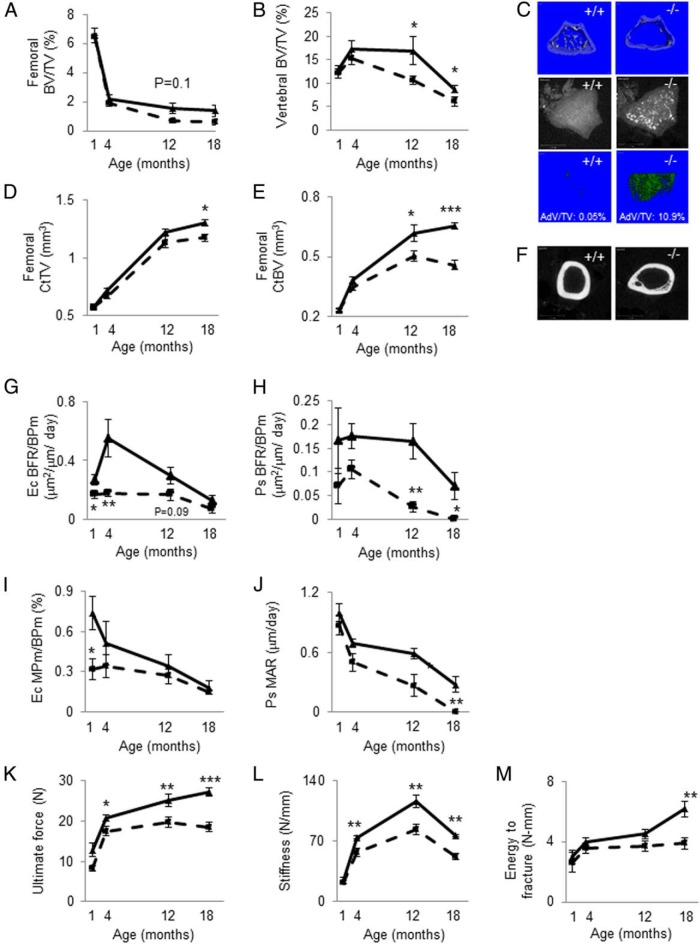
Effect of aging in the absence of PPARβ on bone microarchitecture, bone formation indices, and strength. A, Trabecular BV/TV of femur. B, BV/TV of the vertebral body. C, Upper panel, Illustration of three-dimensional trabecular structure of the distal femur in mice aged 18 months. Middle panel, Two-dimensional illustration of adipocytes stained at the distal femur by osmium and visualized by microcomputed tomography. Lower panel, Three-dimensional reconstruction of adipocyte volume present in the marrow space. CtTV (D) and CtBV (E) at the midshaft femur are shown. F, Two-dimensional reconstruction of the cortical midshaft illustrating the lower CtBV in part by increasing the endocortical porosity. G–J, Bone formation indices at endocortical (Ec) and periosteal (Ps) surfaces. MAR, mineral apposition rate; MPm/BPm, mineralization perimeter on bone perimeter. K–M, Biomechanical properties of the cortical femur obtained by three-point bending tests. *, *P* < .05; **, *P* < .01; ***, *P* < .001 significant difference vs *PPAR*β^−/−^ mice. Continuous lines, *PPAR*β^+/+^; hatched lines, *PPAR*β^−/−^ show means (± SEM).

Age-related changes in trabecular microarchitecture followed a similar pattern in the distal femur and caudal vertebrae, although in the femur the loss of BV/TV occurred earlier and was more dramatic (Figure 2A). At the vertebrae, the decline in BV/TV was higher in *PPAR*β^−/−^ at 12 and 18 months of age (Figure 2B and Supplemental Table 1), whereas trends were observed only at the distal femur. In the same region, ie, the metaphysis of the distal femur, old *PPAR*β^−/−^ mice exhibit higher osmium staining, indicating more bone marrow adiposity (+217% of adipocyte volume per tissue volume vs *PPAR*β^+/+^, *P* < .05) (Figure 2C). In the midshaft femur, cortical tissue volume and bone volume stabilized at the age of 12 months. At 18 months of age, *PPAR*β^−/−^ mice exhibited lower cortical tissue volume and bone volume compared with *PPAR*β^+/+^ mice (Figure 2, D and E) together with increased endocortical porosity (Figure 2F). These changes were associated with higher osteoclast number and surface in *PPAR*β^−/−^ vs *PPAR*β^+/+^ mice (Supplemental Table 1), whereas the bone-forming indices were lower, particularly at the periosteal surfaces at a latter age (Figure 2, G–J). Consistent with bone histomorphometry, at 18 months of age, bone resorption (carboxy-terminal collagen cross-links) and bone formation (osteocalcin) markers were higher and lower, respectively, in *PPAR*β^−/−^ than *PPAR*β^+/+^ mice (Supplemental Table 2), indicating that bone turnover and coupling is altered in the absence of PPARβ. In addition, we found higher circulating levels of myostatin in *PPAR*β^−/−^ mice compared with *PPAR*β^+/+^ mice.

As evaluated by three-point bending, ultimate force and stiffness were lower in *PPAR*β^−/−^ mice compared with *PPAR*β^+/+^ mice at 4, 12, and 18 months (Figure 2, K and L). At 18 months of age, the energy to fracture was also significantly lower in *PPAR*β^−/−^ compared with *PPAR*β^+/+^ mice (Figure 2M).

### Altered osteoblast differentiation in PPARβ-deficient cells

**Figure 3.**
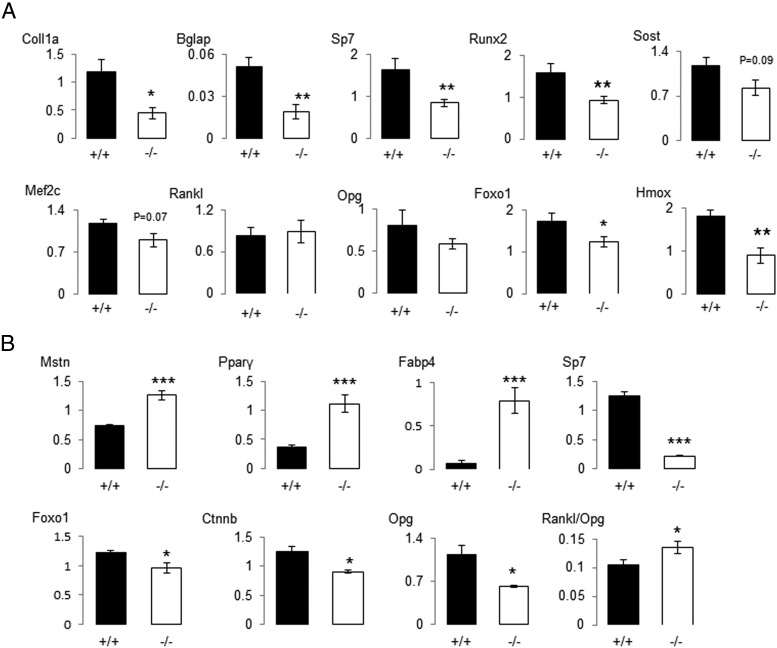
Effect of PPARβ deficiency on gene expression relative to glyceraldehyde-3-phosphate dehydrogenase in the femur of mice aged 18 months (A) and in primary calvarial osteoblasts (B). *Coll1a*, collagen 1a; *Bglap*, osteocalcin; *SP7*, osterix; *Sost*, sclerostin; *Mef2c*, myocyte enhancer factor 2b; *Opg*, osteoprotegerin; *Foxo1*, Forkhead box O1; *Hmox*, hemeoxygenase. *, *P* < .05; **, *P* < .01 significant difference vs *PPAR*β^−/−^ mice. Bars show means (± sem). Closed bars, *PPAR*β^+/+^; open bars, *PPAR*β^−/−^.

To clarify the molecular mechanisms by which *PPAR*β deficiency alters the bone forming activity, we first examined bone mRNA expression levels in the old mice (18 mo of age). Expression levels of genes associated with osteoblast and osteocyte differentiation, as well as *Foxo1* and *Hmox*, were significantly altered in *PPAR*β^−/−^ mice (Figure 3A).

**Figure 4.**
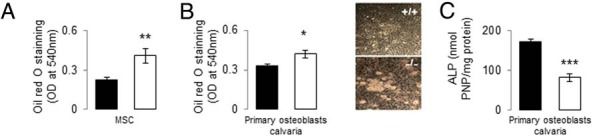
Effect of PPARβ deficiency on MSC differentiation into adipocytic lineage and primary calvarial osteoblasts. A, Oil red O quantification after 14 days of MSCs prepared from *PPAR*β^+/+^ and *PPAR*β^−/−^ bone marrow culture in adipogenic medium. B, Oil red O quantification after 14 days of *PPAR*β^+/+^ and *PPAR*β^−/−^ calvaria culture in adipogenic medium and an illustration of the presence of oil droplets in *PPAR*β^−/−^ calvaria medium. C, Alkaline phosphatase (ALP) production in osteoblasts from *PPAR*β^+/+^ and *PPAR*β^−/−^ mice calvariae after 14 days of culture. *, *P* < .05; **, *P* < .01; ***, *P* < .001 by unpaired *t* test compared with *PPAR*β^+/+^ mice.

*Mstn* as well as *Fabp4* and *Ppar*γ were also up-regulated in calvariae from *PPAR*β^−/−^ mice, ie, independent of weight-bearing and muscle contraction, whereas *Foxo1*, *Ctnnb* (β-catenin), and *Tnsf11b* (*Opg*) gene expression was decreased (Figure 3B). Eventually, cultures of MSCs from *PPAR*β^−/−^ bone marrow showed a low expression levels of *Cbfa1* (−38.3% and −41.0% vs *PPAR*β^+/+^, respectively, after 3 and 7 days of culture, *P* < .05) and an increased propensity to differentiate into adipocytes (Figure 4A). An increased propensity to form mature adipocytes was confirmed in primary osteoblast cultures from *PPAR*β^−/−^ calvariae under adipogenic conditions (Figure 4B) concomitant with lower osteoblast differentiation (Figure 4C). Altogether these data indicate that *PPAR*β deficiency causes a cell-autonomous defect of the osteoblastic lineage.

### Response to physical activity and loading

We first evaluated whether moderate physical activity can restore the musculoskeletal phenotype of *PPAR*β^−/−^ mice, particularly muscle force and bone formation at periosteum surfaces.

**Figure 5.**
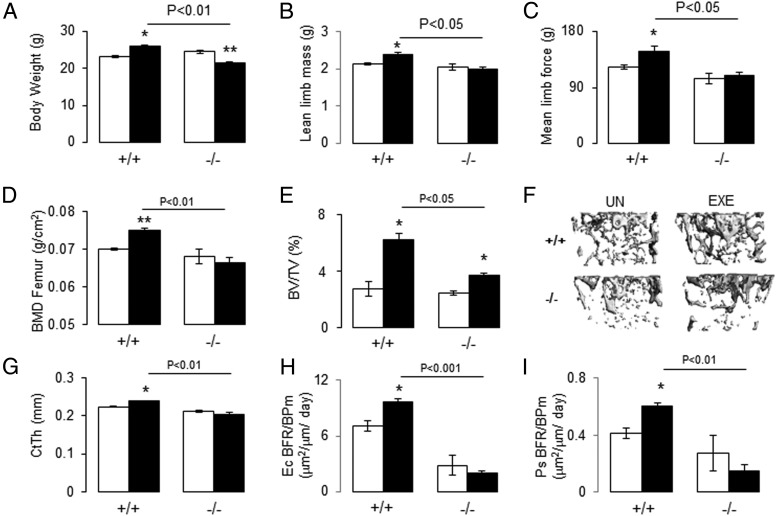
Effect of treadmill exercise on body weight, muscle function, BMD, microarchitecture, and bone formation index in *PPAR*β^−/−^ mice. A, Body weight evaluated by PIXImus (GE Lunar). B, Lean limb mass evaluated by PIXImus. C, Muscle force evaluated by handgrip. D, Femur BMD. E, Trabecular BV/TV. F, Illustration of three-dimensional trabecular structure of the distal femur. G, Cortical thickness (CtTh) at midshaft femur. H and I, Bone formation rate on bone perimeter (BFR/BPm) at endocortical (Ec) and periosteal (Ps) surfaces. *, *P* < .05; **, *P* < .01 significant difference vs sedentary group. Bars show means (±SEM). Closed bars, exercise; open bars, sedentary.

Exercise increased body weight, limb lean mass, and force in *PPAR*β^+/+^ but not in *PPAR*β^−/−^ mice (Figure 5, A–C). Exercise increased trabecular BV/TV in *PPAR*β^+/+^ (+124% vs sedentary, *P* < .05) and to a lesser extent in *PPAR*β^−/−^ mice (+54% vs sedentary, *P* < .05, Figure 5E). However, exercise significantly increased femur BMD and cortical structure (CtBV) in *PPAR*β^+/+^ but not in *PPAR*β^−/−^ mice (Figure 5, D, F, and G). Moreover, exercise stimulated bone formation rate (BFR) at the endocortical and periosteal surfaces in the *PPAR*β^+/+^ but not in the *PPAR*β^−/−^ mice (Figure 5, H and I). These observations therefore indicate an altered muscle and bone anabolic response to exercise in the absence of PPARβ. Of note, exercise significantly reduced glucose area under the curve in both *PPAR*β^+/+^ and *PPAR*β^−/−^ mice (Supplemental Figure 1B).

**Figure 6.**
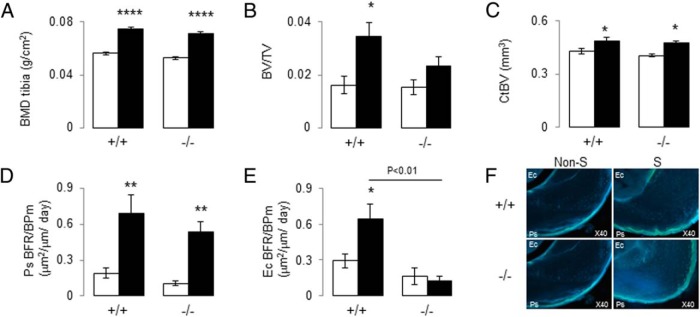
Effect of axial compression on BMD, microarchitecture, and bone formation index in *PPAR*β^−/−^ mice. A, Tibia BMD. B, Trabecular BV/TV of the proximal tibia. C, CtBV at the midshaft tibia. D and E, Bone formation rate on bone perimeter (BFR/BPm) at endocortical (Ec) and periosteal (Ps) surfaces. F, Fluorescent calcein labels on transverse cortical midshaft tibia. *, *P* < .05; **, *P* < .01; ****, *P* < .0001 significant difference vs nonstimulated. Bars show means (±SEM). Closed bars, stimulated tibia (S); open bars, nonstimulated tibia (Non-S).

Having established that PPARβ modulates the muscle and bone response to moderate physical activity, we next asked whether the lack of skeletal response in *PPAR*β^−/−^ was secondary to the muscle dysfunction and/or could be explained by an intrinsic failure of the bone mechanotransduction. To this aim, mice were subjected to direct axial compression of the tibia in vivo for 2 weeks. Contrasting with exercise, axial compression of the tibia significantly increased BMD and CtBV in both *PPAR*β^+/+^ and *PPAR*β^−/−^ mice (Figure 6, A–C). Loading also stimulated periosteal bone formation similarly in *PPAR*β^+/+^ and *PPAR*β^−/−^ mice (Figure 6, D and F). However, at the endocortical surface, loading increased the bone-forming indices only in *PPAR*β^+/+^ mice (Figure 6, E and F). Taken together, these data indicate that PPARβ deficiency alters the periosteal modeling response induced by muscle activity but not by direct mechanical loading, whereas alterations of endosteal bone remodeling occur independently of muscle activity.

## Discussion

Our results establish several important new findings regarding the role of PPARβ in the pathogenesis of bone fragility in diabetes. First, in the absence of PPARβ, there is an accelerated and parallel decline of muscle strength and bone formation, predominantly cortical, with age, leading to lower mechanical properties (bone strength). Interestingly, mechanical deterioration occurs before cortical structure changes are observed, consistent with the notion of early alterations in the material properties of the bones in these mice. Second, exercise was not able to improve muscle and bone parameters in *PPAR*β^−/−^ mice, whereas direct mechanical loading partially was successful to this end by improving periosteal bone formation, thereby suggesting that PPARβ is involved in the reduced ability of diabetic bone to withstand mechanical loading, particularly at remodeling surfaces. Third, PPARβ deficiency led to increased *PPAR*γ expression in vivo and in vitro, increased differentiation of MSCs into adipocytes, and increased bone marrow adiposity, thereby providing a new mechanism to explain the reduced bone formation in relation to glucose intolerance and lipid accumulation.

The metabolic syndrome and T2DM are characterized by a compromised ability of insulin to control glucose disposal in muscle, increased hepatic glucose production, and overt postprandial hyperglycemia. In accordance with the literature, our study confirms that *PPAR*β^−/−^ mice are metabolically less active and glucose intolerant from young adulthood to older age (11). In our study the glucose intolerance was not significant at 12 months of age, but we still observed the same trend as at an earlier age. This was probably due to an artifact of glucose measurement or mice not completely fasted because *PPAR*β^−/−^ mice are again intolerant at 18 months of age. We specifically provide three lines of evidence indicating that PPARβ is also implicated in the pathogenesis of bone fragility that could be observed in metabolic syndrome.

First, PPARβ deficiency not only alters the proportion of type II/type I fibers and muscle oxidative function (28) but also plays a major role in the maintenance of muscle mass and strength during aging. These two latter factors are now recognized as significant determinants of bone fragility and frailty (29, 30). Most prominently, PPARβ deficiency increased myostatin levels both locally and systemically, which can negatively impact on both muscle development and osteoblastic function (31–33). Indeed, in vitro, PPARβ activation induces myogenesis by inhibiting myostatin expression (34). Interestingly, type 2 diabetic patients have an up-regulated plasma myostatin associated with increased body mass index, higher fasting plasma glucose, and blood insulin sensitivity (35, 36). Myostatin and its receptors, type IIA and IIB activin Rc, have become prominent targets for the development of therapeutic inhibitors (37), with the potential for use in muscle dysfunction associated with T2DM and sarcopenic patients (now in phase II-III clinical trials, see http://clinicaltrials.gov/ct2/results?term=myostatin&Search=Search). Thus, increased myostatin expression in PPARβ-deficient mice may at least partly explain their poor muscle strength.

Second, in bone, PPARβ has been shown to regulate *Opg* expression and osteoclastogenesis through the β-catenin signaling pathway (20). We also observed decreased *Ctnnb* and *Opg* expression in PPARβ-deficient cells and an increase in osteoclast number and bone resorption markers in *PPAR*β^−/−^ mice. In accordance, we observed the appearance of cortical porosity in *PPAR*β^−/−^ mice. Furthermore, our data demonstrate that PPARβ in bone cells plays a role in the regulation of osteoblast differentiation and function with lower expression of *Collagen 1a*, *Runx2*, and *Osterix*.

We also show an alteration of osteoblastic oxidative stress in the absence of PPARβ, as previously demonstrated in myoblasts, endothelial cells, and the ROS17/2.8 osteoblastic cell line (38–40). In vivo our results indicate that bone formation indices are negatively altered at the endocortical surfaces at early time points and later on at the periosteum. Consistent with this, *PPAR*β^−/−^ exhibits a lower BMD and cortical structure, ie, CtTV and CtBV in adult and old mice. With regard to the biomechanical properties, the femurs of the knockout mice exhibit lower ultimate force, stiffness, and energy to fracture. Interestingly, young adult *PPAR*β^−/−^ mice exhibit a degradation of biomechanical properties without any significant changes in BMD and microarchitecture, suggesting an alteration of bone material properties. Previous rodent models suggest that a dysregulation of glucose metabolism leads to an accumulation of reactive oxygen species such as advanced glycation end-products and superoxide dismutase, which interact with bone matrix collagen and therefore change the mechanical properties of the bone (10). This has been demonstrated in Zucker rats and yellow Kuo Kondo mice (41); however, all these models exhibit a low BMD. Therefore, *PPAR*β^−/−^ mice are unique in that they display similar key metabolic and skeletal characteristics as in human T2DM patients, namely lower mechanical properties and higher cortical porosity in the absence of low BMD (5).

In addition *PPAR*β^−/−^ mice were characterized by a higher fat infiltration in the bone marrow, which can also influence bone quality. This fat accumulation was associated with an up-regulation of PPARγ expression. In vitro, we confirm a higher transdifferentiation activity of *PPAR*β^−/−^ MSCs into adipocytes, paralleled by an overexpression of PPARγ. Activation of the PPARγ2 isoform leads to MSC differentiation toward the adipocyte lineage at the expense of the osteoblast (42). Importantly, there has been increasing evidence that the inverse correlation between bone and fat phenotypes also exists in osteoporosis (42) and T2DM patients (43).

Third, exercise was unable to restore muscle and/or bone mass and strength in *PPAR*β^−/−^ mice, contrasting with preclinical and clinical studies showing an improvement of muscle function by exercise through an increase in glycogen synthesis and a decrease in lipid accumulation in rodent models of type 2 diabetes (44, 45) and T2DM subjects (46, 47). In contrast, direct mechanical loading of the tibiae restored periosteal bone formation but not the endocortical response. We hypothesize that the lack of periosteal bone response to exercise in *PPAR*β^−/−^ may be attributed to lower muscle contraction in these mice because muscle stimulation is not involved in the bone response to direct mechanical loading. This explanation is consistent with reports in the literature describing lower muscle function correlating with reduced PPARβ expression (48, 49), with the lower grip strength observed in the *PPAR*β^−/−^ mice and with the known effects of muscle contraction on bone structure (50). Alternatively, the lack of PPARβ in muscle may have altered the expression of a bone growth factor such as *Igf-1*, bone morphogenetic protein (*Bmp*), and/or other myokines, known to be involved in the musculoskeletal system in response to exercise (51, 52). In our model, *Igf-1* is not significantly affected; however, *Bmps* and *Mstn* are expressed, respectively, less and more in *PPAR*β^−/−^ mice compared with their wild-type littermates. Thus, further studies aimed at investigating whether the use of a myostatin inhibitor or BMP treatment can restore the bone response to exercise of *PPAR*β^−/−^ mice are warranted.

To potentially explain the absence of bone formation at the endocortical surfaces in loaded *PPAR*β^−/−^ mice, two major hypotheses are suggested. First, the absence of *PPAR*β may alter the structure of the bone matrix, particularly at the endocortical surface, and therefore impair its mechanotransduction properties, as we previously reported for the periosteal surfaces in periostin-deficient mice (26). Second, bone marrow stem cells are capable of sensing exogenous mechanical signals and shift MSC differentiation toward osteoblastogenesis and away from adipogenesis (53, 14). These alterations have been related to decreased *Runx2* and increased *PPAR*γ expression, respectively (19), two transcriptions factors modulated by PPARβ. Therefore, the absence of a bone response to axial compression at the endocortical compartment could be attributed to the default of MSCs to differentiate into the osteoblastic lineage.

In conclusion, the absence of PPARβ leads to three major systemic changes, namely glucose intolerance, decreased muscle strength, and exercise capacity, together with low bone formation and response to exercise. At the molecular level, PPARβ appears to regulate myostatin and *PPAR*γ levels in muscle and bone, thereby providing potential new mechanisms to explain bone fragility related to metabolic syndromes and potentially identifying new molecular targets for the treatment of muscle and bone fragility in diabetes.
